# Intraoperative Contamination of Sterile Fields and Postoperative Implications in Total Hip and Knee Arthroplasty: A Prospective Observational Study

**DOI:** 10.3390/jcm15082986

**Published:** 2026-04-14

**Authors:** Nicolas Catalin Ionut Ion, Sorin Radu Fleaca, Bogdan Axente Bocea, Cosmin-Ioan Mohor, Mihai-Dan Roman, Calin-Ilie Mohor, Alexandru Florin Diconi, Alexandru Turcu, Vicentiu Vasile Veres, Iustin-Ilie Tutuianu, Mihai Faur, Vanesa-Maria Veres, Victoria Birlutiu

**Affiliations:** 1 Faculty of Medicine, Lucian Blaga University of Sibiu, Str. Lucian Blaga, 550169 Sibiu, Romania; nicolascatalinionut.ion@ulbsibiu.ro (N.C.I.I.); bogdanaxente.bocea@ulbsibiu.ro (B.A.B.); cosmin.mohor@ulbsibiu.ro (C.-I.M.); mihairoman@ulbsibiu.ro (M.-D.R.); calin.mohor@ulbsibiu.ro (C.-I.M.); alexandru.diconi@ulbsibiu.ro (A.F.D.); alexandruss.turcu@ulbsibiu.ro (A.T.); vicentiu.veres@ulbsibiu.ro (V.V.V.); iustin.tutuianu@ulbsibiu.ro (I.-I.T.); mihai.faur@ulbsibiu.ro (M.F.); vanesa.veres@ulbsibiu.ro (V.-M.V.); victoria.birlutiu@ulbsibiu.ro (V.B.); 2 County Clinical Emergency Hospital of Sibiu, 550245 Sibiu, Romania; 3 County Clinical Emergency Hospital of Cluj-Napoca, 010024 Cluj-Napoca, Romania

**Keywords:** joint, infection, periprosthetic, staphylococcus, intraoperative

## Abstract

**Introduction:** Periprosthetic joint infections (PJI) are among the most serious and costly complications in orthopedic surgery, significantly affecting patient prognosis and healthcare systems. Despite rigorous aseptic measures, intraoperative contamination of sterile fields, instruments, and air remains a persistent source of potential infection. This study investigates the relationship between the microbial contamination of sterile fields during arthroplasty and postoperative inflammatory markers, with the objective of determining whether the contamination of sterile fields correlates with the presence of periprosthetic joint infection (PJI). **Material and Methods:** This prospective observational study included 33 patients undergoing total hip or knee arthroplasty in a university-affiliated orthopedic center. Intraoperative samples were collected from sterile fields and equipment to detect microbial contamination, while postoperative monitoring involved the C-reactive protein (CRP); erythrocyte sedimentation rate (ESR); leukocyte count; temperature; and wound assessment on days 1, 3 and 7. All patients received 48 h of prophylactic cefuroxime. Statistical analysis was conducted using the International Business Machines (IBM) Statistical Product and Service Solutions (SPSS) software for Windows, version 30.0 (IBM Corporation, Armonk, New York, United States of America) with significance set at *p* ≤ 0.05. **Results:** Postoperative inflammatory markers showed distinct patterns depending on the isolated microorganism, with Proteus vulgaris and *Staphylococcus hominis* ssp. consistently associated with higher CRP and leukocyte values, indicating a more intense systemic response. Staphylococcus epidermidis was the most frequently isolated species but showed moderate inflammatory profiles, suggesting its potential role in subclinical colonization. A strong correlation between CRP on day 3 and leukocyte count (r = 0.81) confirms their combined utility in the early detection of infectious complications, while ESR appeared less dynamic and more complementary in nature. **Discussion**: This study highlights the significant role of intraoperative contamination and microbial virulence in shaping the postoperative inflammatory response after arthroplasty. Elevated CRP and leukocyte levels, particularly on day 3, were closely associated with pathogens known for biofilm formation and chronic infections. Despite prophylactic antibiotic use, confirmed infections still occurred, suggesting the need to reassess current protocols and enhance intraoperative contamination control. **Conclusions:** Pathogen presence in sterile fields during arthroplasty increases the risk of periprosthetic joint infections, often without early clinical symptoms. CRP on day 3 and leukocyte count were the most reliable early indicators of persistent inflammation.

## 1. Introduction

Periprosthetic joint infections represent one of the most severe and costly complications in orthopedic surgery [1], with major impacts on patient outcomes and healthcare systems. Despite the implementation of strict aseptic protocols during surgical interventions, studies have shown that intraoperative contamination of the environment (sterile fields, instruments, air) continues to be a potential source of bacterial colonization and delayed infection [2,3,4].

Bacterial contamination can occur despite visible cleanliness, and the microbial load in the operating room air, equipment, and critical areas is directly correlated with the incidence of postoperative infections [5]. The presence of microorganisms in samples collected from surfaces considered sterile—such as instrument tables, surgical lamps, and drapes—has been documented in multiple studies, indicating that contamination sources may be endogenous (the patient) or exogenous (personnel, equipment, air) [6,7].

Numerous studies have demonstrated that factors such as the number of people in the operating room, the frequency of door openings, and noncompliance with gowning protocols directly contribute to the increase in airborne particulate matter capable of carrying bacteria [8]. Additionally, some bacterial strains isolated intraoperatively are capable of forming biofilms, which makes them difficult to detect and treat later [9].

This study aims to analyze postoperative inflammatory parameters and the clinical occurrence of infections based on the presence of microorganisms identified during total hip and knee arthroplasty procedures.

## 2. Materials and Methods

This is a prospective observational study conducted in a university-affiliated orthopedic center. We analyzed a cohort of 33 patients with chronic conditions who underwent total hip and knee arthroplasty over a 2 month period. Patients were eligible for inclusion if they were adults (≥18 years) undergoing primary total hip arthroplasty (THA) or total knee arthroplasty (TKA) at the study center during the study period. All procedures were performed in a controlled operating room environment following standard aseptic orthopedic surgical protocols. Exclusion criteria included: revision arthroplasty, pre-existing joint infection, active systemic infection at the time of surgery, immunosuppressive therapy, and refusal to provide informed consent.

According to prior findings, multiple pathogenic agents were identified intraoperatively in sterile surgical fields. To evaluate the presence of pathogens in the operating room environment, samples were collected using tubes containing Amies Viscosa transport medium at three time points: at the beginning of the surgery, 60 min after the procedure started, and at the end of the operation. Sampling was performed from several sterile surfaces, including the sterile drape in the incision area, located 10 cm from the surgical incision on the sterile isolation field (hip/knee arthroplasty surgery pack, Submed), as well as 50 cm from the same sterile field. Additional samples were obtained from the handles of the surgical lighting lamps and from the instrument table. In the laboratory, samples were inoculated onto standard culture media, including blood agar and MacConkey agar, and incubated under aerobic conditions at 35–37 °C for 24–48 h. In cases of suspected slow-growing organisms, incubation was extended up to 72 h. Bacterial identification was performed using conventional biochemical methods and/or automated identification systems, in accordance with routine laboratory protocols. No molecular diagnostic techniques (e.g., PCR or sonication fluid analysis) were available in this study, which may have limited the detection of low-grade or biofilm-associated infections.

During surgery, we monitored the number of personnel in the operating room as well as the frequency of door openings. Postoperative monitoring included the collection of inflammatory markers—C-reactive protein (CRP), erythrocyte sedimentation rate (ESR), and white blood cell count—on postoperative day 1, day 3 and day 7. CRP, ESR, and leukocyte count were used to monitor postoperative inflammatory response dynamics; however, these markers were not considered specific for infection and were not used as standalone diagnostic criteria for periprosthetic joint infection. Each patient’s temperature was recorded to detect febrile episodes. Wound dressings were changed daily to assess for exudate or signs of local infection. The diagnosis of periprosthetic joint infection (PJI) was assessed according to the criteria proposed by the International Consensus Meeting (ICM) and the Musculoskeletal Infection Society (MSIS). These criteria incorporate clinical signs, microbiological findings, and laboratory markers to establish the presence of infection. In the present study, the diagnosis of suspected postoperative infection was based on the dynamic evaluation of inflammatory markers (CRP, ESR, leukocyte count), clinical signs such as fever or wound abnormalities, and microbiological findings from intraoperative samples. Intraoperative contamination was defined as the isolation of microorganisms from samples collected from presumed sterile fields or surgical equipment. This finding was interpreted as environmental or procedural contamination and not as evidence of infection. Colonization was considered in cases where microorganisms were detected without significant elevation in inflammatory markers or clinical signs of infection.

All patients received prophylactic antibiotic therapy consisting of cefuroxime (a second-generation cephalosporin) administered three times daily for 48 h, which is commonly used for the treatment of various bacterial infections. Statistical analysis was performed using the International Business Machine (IBM) Statistical Product and Service Solutions (SPSS) software package for Windows, version 30.0 (IBM Corporation, Armonk, New York, United States of America). A *p*-value of ≤ 0.05 was considered statistically significant for all analyses. Correlation analysis between inflammatory markers was performed using Spearman’s rank correlation coefficient due to the non-normal distribution of several variables, as confirmed by the Shapiro–Wilk test.

## 3. Results

**Figure 1:**
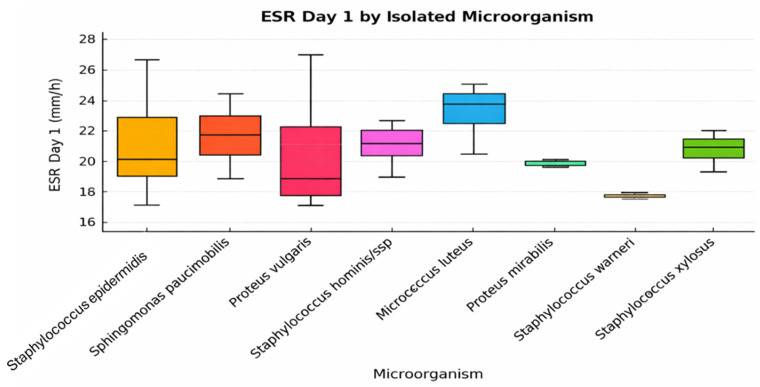
Distribution of ESR (Day 1) across different isolated microorganisms.

A total of 18 patients underwent knee arthroplasty, while the remaining 15 underwent hip arthroplasty. The chart illustrates the variations in ESR on postoperative day 1 based on the isolated microorganism. Compared with leukocyte counts, which demonstrated relatively limited variability, ESR values (Figure 1) exhibited greater dispersion across patients, indicating a more heterogeneous inflammatory response depending on the isolated microorganism. Higher individual ESR values were observed in some cases involving *Staphylococcus epidermidis* and *Micrococcus luteus* (Figure 1); however, these observations are descriptive and do not imply statistically significant differences between microorganism groups.

*Staphylococcus hominis* ssp. and *Sphingomonas paucimobilis* also exhibit moderate to high values, suggesting a systemic inflammatory response present as early as the first postoperative day (Figure 1). In contrast, *Proteus mirabilis*, *Staphylococcus warneri*, and *Staphylococcus xylosus* are associated with lower ESR values and reduced variability. This may indicate colonization rather than active infection or localized inflammation. The larger dispersion observed in cases associated with *Proteus vulgaris* may reflect the heterogeneous host inflammatory response to Gram-negative pathogens and the relatively small sample size of this subgroup. Gram-negative bacteria are known to induce variable systemic responses through endotoxin-mediated immune activation. Conversely, certain microorganisms showing minimal inflammatory marker elevation may represent low-virulence colonization rather than clinically significant infection.

**Figure 2:**
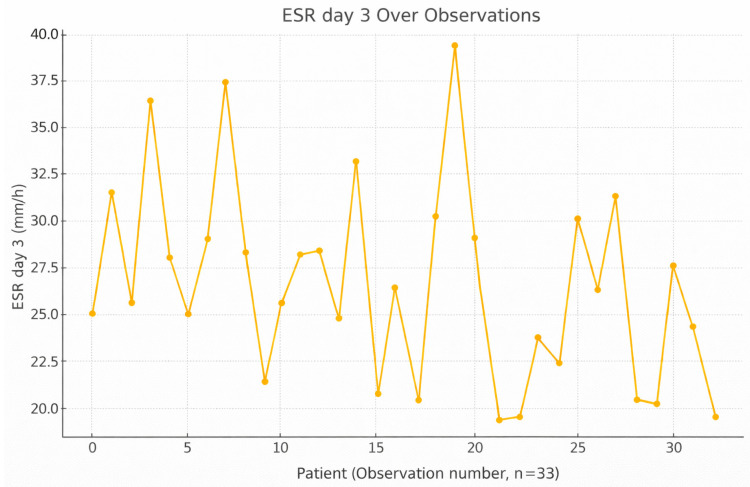
Variation and trend of ESR levels on day 3 across individual observations.

By postoperative day 3, ESR values showed a variable pattern across patients, with a general tendency toward higher values compared with day 1, indicating the body’s ongoing inflammatory reaction to surgical stress or infection; however, no statistically significant difference was observed (Figure 2). Unlike CRP, ESR rises more slowly but stays elevated longer, making it a complementary marker. ESR correlates moderately with CRP and varies by pathogen virulence, with *Staphylococcus epidermidis* and *Sphingomonas paucimobilis* associated with higher values. This time point helps capture the mid-phase of inflammation, where resolution or escalation can be monitored.

**Figure 3:**
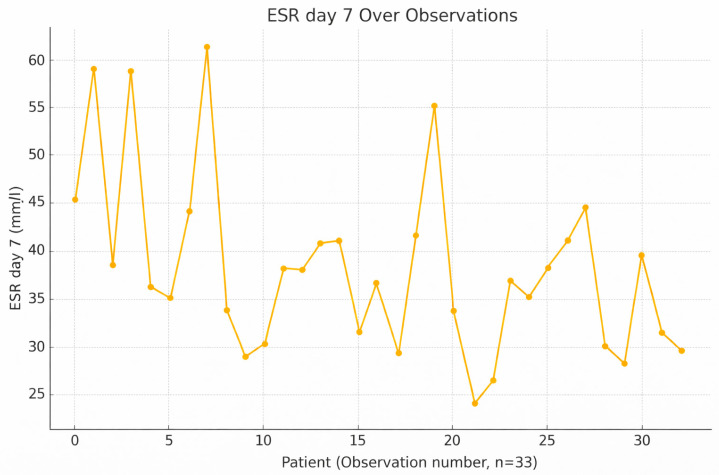
Variation and trend of ESR levels on day 7 across individual observations.

ESR values often peak around day 7, as they continue to reflect long-term inflammatory status even after CRP starts declining (Figure 3). This is particularly important in assessing chronic inflammation or slow recovery, as ESR remains elevated despite short-term improvement. ESR is not a standalone marker but adds value when interpreted with CRP and leukocyte data. A high ESR at this stage may not indicate acute infection but should still be evaluated in context.

It is important to note that ESR is a marker with a slower response time compared with CRP, and its interpretive value increases when assessed dynamically over time rather than at a single time point.

**Figure 4:**
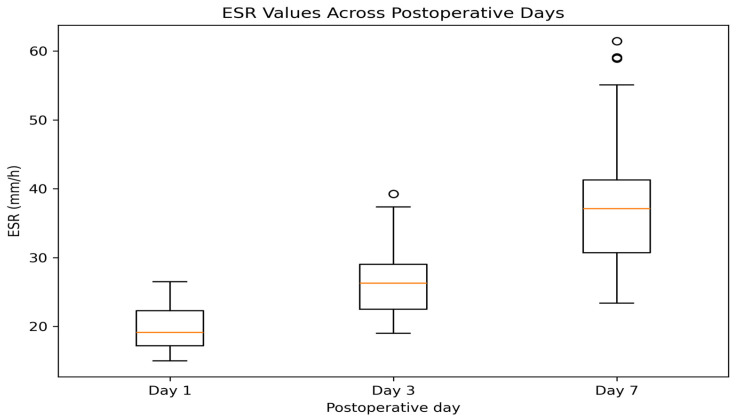
Distribution of erythrocyte sedimentation rate (ESR) on postoperative days 1, 3, and 7.

A gradual increase in ESR is observed across the three time points, with the highest median values and dispersion on day 7 (Figure 4). This pattern is consistent with the slower kinetic profile of ESR compared with CRP, reflecting a more prolonged inflammatory response.

**Figure 5:**
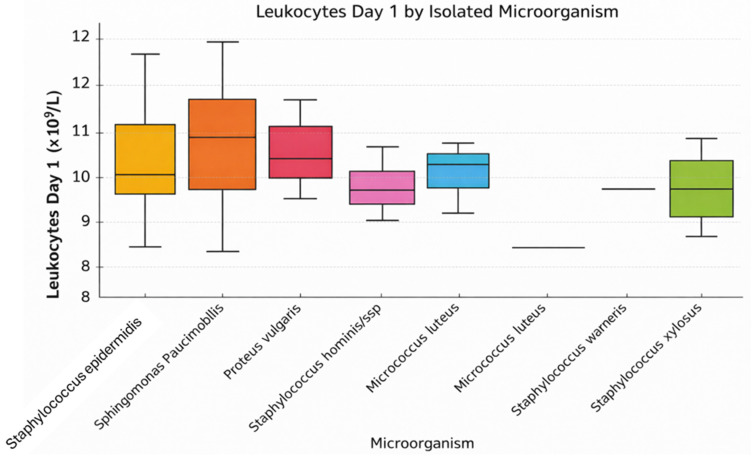
Distribution of leukocyte counts (Day 1) across different isolated microorganisms.

Figure 5 illustrates leukocyte values on postoperative day 1 according to the intraoperatively isolated microorganism. It is observed that patients infected with *Staphylococcus epidermidis*, *Sphingomonas paucimobilis*, and *Proteus vulgaris* tend to have higher leukocyte counts compared with other groups. The medians are around 10 × 10^9^/L, with some notable variations.

For example, *Proteus vulgaris* and *Sphingomonas paucimobilis* show slightly elevated values, suggesting a more intense immune response within the first 24 h. In contrast, microorganisms such as *Staphylococcus hominis* ssp. and *Staphylococcus xylosus* are associated with lower leukocyte values and reduced variability. Additionally, there are a few outliers for *Staphylococcus epidermidis*, indicating possible severe systemic infections in certain cases. These observations support the role of leukocytes as an early biomarker, though insufficient when used in isolation, for assessing the severity of postoperative inflammation or infection.

**Figure 6:**
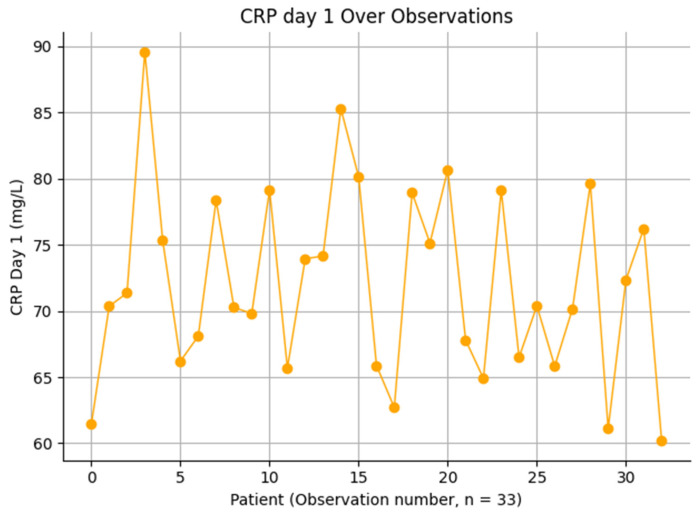
Variation and trend of C-reactive protein (CRP) Levels on Day 1 Across Individual Observations.

CRP values on postoperative day 1 demonstrated moderate variability across patients, with most values ranging between 60 and 90 mg/L (Figure 6). The distribution appears relatively homogeneous, without extreme outliers, suggesting a consistent early inflammatory response following surgical intervention. This pattern likely reflects the physiological response to surgical trauma rather than infection-specific processes. The absence of marked dispersion or extreme elevations supports the limited diagnostic value of CRP in the immediate postoperative period.

**Figure 7:**
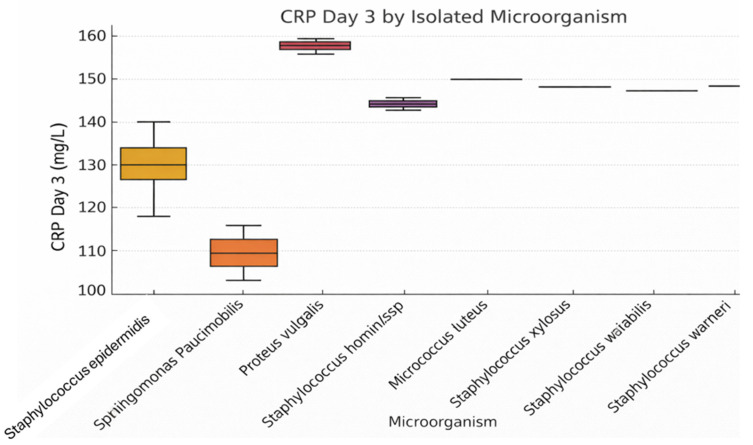
Distribution of CRP Levels (Day 3) by Isolated Microorganism.

Figure 7 analyzes CRP values on postoperative day 3 according to the intraoperatively isolated microorganism. Significantly elevated levels are observed for *Proteus vulgaris* and *Staphylococcus hominis* ssp., indicating a marked systemic inflammatory response. Although *Staphylococcus epidermidis* is the most frequently isolated organism, CRP levels remain relatively moderate. The chart suggests that the severity of inflammation is not solely a function of bacterial presence but also depends on the virulence of the pathogenic agent.

**Figure 8:**
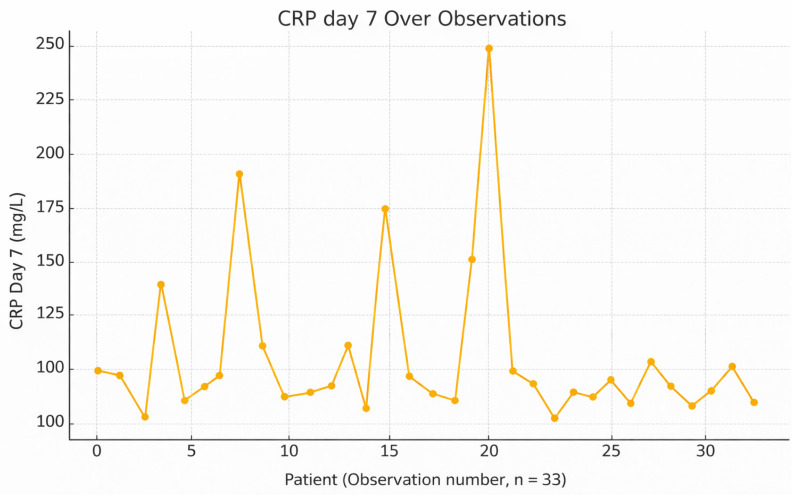
Variation and *trend* of C-*reactive protein* (CRP) levels on day 7 across individual observations.

On postoperative day 7, CRP values generally declined, reflecting a typical resolution of the early acute inflammatory response (Figure 8). However, elevated CRP in some patients may suggest a persistent infection, especially with pathogens like *Proteus vulgaris* or *Staphylococcus hominis* ssp., known for inducing stronger inflammatory reactions. Since CRP responds rapidly to treatment, continued elevation at this stage should prompt clinical reassessment. CRP on day 3 correlates strongly with systemic inflammation and leukocyte count.

**Figure 9:**
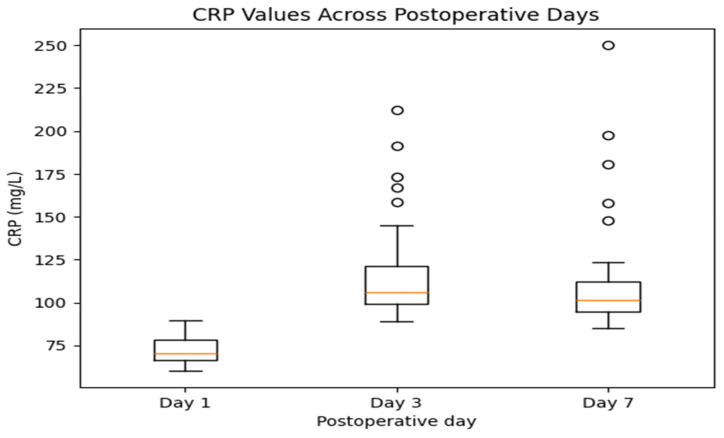
Distribution of C-reactive protein (CRP) levels on postoperative days 1, 3, and 7.

CRP levels show an increase from day 1 to day 3, followed by a tendency toward stabilization or decline by day 7. The highest variability is observed on day 3, consistent with the expected peak of the postoperative inflammatory response within 48–72 h. While most patients demonstrate decreasing values by day 7, a subset maintains elevated CRP levels, which may reflect ongoing inflammatory activity (Figure 9).

**Figure 10:**
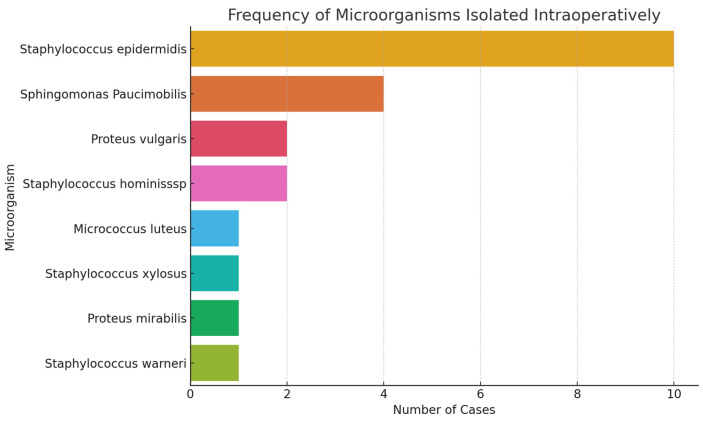
Frequency of microorganisms isolated during intraoperative sampling.

Figure 10 provides an overview of the frequency of microorganisms isolated intraoperatively. *Staphylococcus epidermidis* is by far the most frequently detected agent, followed by *Sphingomonas paucimobilis* and *Proteus vulgaris*. The remaining species appear sporadically, which may suggest either incidental contamination or lower pathogenicity. This microbiological profile may be valuable for establishing personalized antibiotic prophylaxis strategies.

The Shapiro–Wilk test demonstrated that most variables, including CRP on postoperative days 1, 3, and 7, as well as ESR on day 7, did not follow a normal distribution (*p* < 0.05). In contrast, ESR values on postoperative days 1 and 3 were normally distributed (*p* > 0.05) (Table 1).

Descriptive statistics of inflammatory markers are presented in Table 2. CRP values peaked on postoperative day 3 (mean 118.05 mg/L) and remained elevated on day 7 (mean 113.06 mg/L), while ESR values showed a gradual increase from day 1 to day 7. Median values were consistently lower than means for CRP.

**Figure 11:**
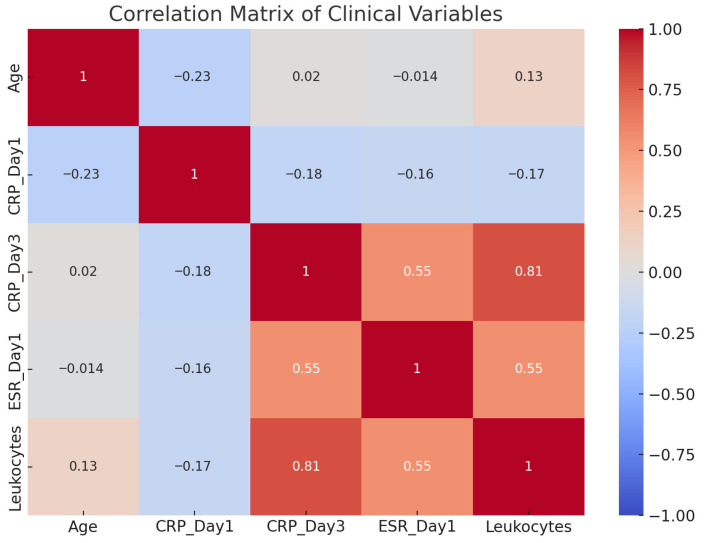
Correlation matrix depicting relationships among age, inflammatory markers (CRP and ESR), and leukocyte counts in the study population.

The presented correlation matrix offers a synthetic analysis of the relationships between postoperative biological markers (CRP on day 1 and day 3, ESR, and leukocytes) and patient age (Figure 11). A strong positive correlation between CRP on postoperative day 3 and leukocyte count was identified using Spearman’s rank correlation analysis (r = 0.81), indicating a consistent association between these markers. Additionally, CRP on day 3 shows a moderate correlation with ESR (r = 0.55), supporting the hypothesis that ESR reflects a longer-term inflammatory process with slower dynamics compared with CRP.

The relationships between CRP on day 1 and the other markers are weak, with slight negative correlations, suggesting that the initial CRP value may not be a sufficient predictor of later inflammatory progression. Patient age does not exhibit significant correlations with any of the inflammatory parameters, indicating a biological independence between the inflammatory response and chronological age in this context.

The matrix confirms that a combined analysis of CRP on day 3 and leukocyte count provides a more accurate picture of the intensity of the inflammatory process and may be useful for early screening of infectious complications. The moderate correlations between ESR and the other markers reinforce its role as a complementary, though not standalone, indicator in the evaluation of postoperative inflammation.

During the postoperative follow-up period, the majority of patients had an uncomplicated clinical course. A subset of patients demonstrated elevated inflammatory markers without clear clinical signs of infection, which were interpreted as a postoperative inflammatory response. Suspected infection was identified in a limited number of cases based on clinical and laboratory findings.

## 4. Discussion

Intraoperative contamination during arthroplasty procedures represents a major risk factor for the development of PJI, one of the most severe complications in orthopedic surgery. The results of this prospective observational study highlight key aspects related to the dynamics of inflammatory markers in relation to the type of microorganism isolated, the efficacy of antibiotic prophylaxis, and the possible presence of subclinical infections sustained by bacterial biofilm.

CRP and leukocyte count are widely used markers for monitoring postoperative inflammation; however, they lack specificity for infection, particularly in the early postoperative period. Following arthroplasty, CRP levels typically peak within 48–72 h as part of the normal inflammatory response to surgical trauma, and leukocytosis may occur independently of infection. Therefore, elevations in these markers must be interpreted with caution and in conjunction with clinical findings and standardized diagnostic criteria. In this study, these biomarkers were used to assess inflammatory trends rather than to establish the presence of periprosthetic joint infection.

The data show a strong correlation between CRP values on postoperative day 3 and leukocytosis, indicating a sustained systemic inflammatory response. In particular, microorganisms such as *Proteus vulgaris*, *Staphylococcus hominis* ssp., and *Micrococcus luteus* were associated with elevated CRP and leukocyte levels, suggesting either increased virulence or the ability to form biofilm. These findings are consistent with the literature, which emphasizes the role of these pathogens in chronic low-grade infections [10]. Although a strong correlation was observed between CRP on postoperative day 3 and leukocyte count, this finding should be interpreted with caution. Both markers are known to increase in response to a wide range of inflammatory stimuli, including surgical trauma, and therefore lack specificity for infection. The concurrent elevation in CRP and leukocyte count likely reflects the overall magnitude of the systemic inflammatory response rather than the presence of periprosthetic joint infection. Consequently, this correlation should not be considered indicative of diagnostic value but rather as a marker of inflammatory activity.

This observation underscores the importance of integrating biological markers with clinical findings and standardized diagnostic criteria, such as MSIS/ICM definitions, when evaluating suspected infection.

A relevant observation in this study is the high frequency of *Staphylococcus epidermidis* isolation—a common skin flora organism known for its ability to form biofilms on prosthetic surfaces [11]. Although not consistently associated with elevated inflammatory markers, its presence must be interpreted with caution, as it may contribute to subclinical infections and late prosthetic failures. Parvizi et al. (2011) reported that periprosthetic infections caused by coagulase-negative staphylococci are frequently underdiagnosed due to subtle clinical manifestations and delayed activation of inflammatory markers [12].

Another significant aspect is that prophylactic use of cefuroxime did not entirely prevent the occurrence of confirmed infections, raising concerns about the adequacy of standard antibiotic regimens in the current context of increasing antimicrobial resistance. This concern is supported by recent guidelines from the IDSA (2020) [13], which recommend re-evaluating standard protocols based on the local microbiological spectrum and individual patient risk profiles [14]. In our study, alternative antibiotics were used in a small number of cases but with a relatively higher proportion of confirmed infections, suggesting either a selection bias toward higher-risk patients or reduced efficacy of the chosen antibiotic regimen.

The distribution of isolated microorganisms supports the hypothesis of predominantly endogenous, skin-derived contamination—an idea reinforced by the work of Arciola et al. (2018) [15], who emphasize the importance of proper skin disinfection, sterile gowning techniques, and laminar airflow systems to control airborne contamination [16]. In this context, the re-evaluation of preoperative disinfection protocols and enhanced training of surgical personnel are essential. It is not sufficient to apply standardized procedures alone; ensuring a high level of compliance and continuous monitoring of operating room practices is crucial.

The correlation matrix of biological parameters revealed significant relationships between CRP on day 3 and other markers, especially leukocytes and ESR. These correlations underscore the value of CRP as a dynamic marker for monitoring systemic postoperative inflammation. The results align with the findings of Glehr et al. (2013) [17], which indicate that a persistent increase in CRP during the first 3–5 postoperative days is strongly suggestive of an infectious complication. In contrast, CRP values on day 1 did not show significant correlations, likely due to the immediate surgical trauma rather than an infectious process.

Another critical issue addressed is the presence of subclinical infections. These are difficult to diagnose and often overlooked in routine clinical practice. Biofilms formed on implants allow bacteria to persist in a low metabolic state, shielded from the host immune response and antibiotic action. Detecting them requires advanced methods such as sonication [18,19], multiple tissue cultures, or quantitative PCR—methods that were not available in this study. This limitation should be acknowledged and represents an opportunity for future developments in the microbiological diagnosis of PJI [19,20].

Regarding chart interpretation, the analysis of CRP distribution on day 3 shows significant differences between microorganisms, suggesting that each pathogen contributes differently to the severity of the inflammatory response [21]. This finding confirms that not all contaminations carry the same infectious potential and that integrating clinical, microbiological, and biological data is essential for therapeutic decision-making. Additionally, the graph depicting infection distribution based on postoperative antibiotic regimen raises legitimate questions regarding the adaptability of current therapeutic protocols to the modern bacterial resistance profile [22,23,24].

The graphical analyses offer significant insights into the temporal dynamics of postoperative inflammation following arthroplasty. These biomarkers, when interpreted together, enhance the diagnostic value for identifying complications such as PJI.

CRP typically peaks within the first 48–72 h post-surgery and should decline steadily by day 7 in uncomplicated cases. However, in this study, a subset of patients maintained elevated CRP values on day 7, raising concern for persistent inflammation or subclinical infection. This is consistent with the literature indicating that prolonged CRP elevation correlates with poor infection control or ineffective antibiotic coverage [25,26]. Therefore, CRP day 7 should be viewed not only as a recovery marker but also as a red flag in cases of stagnating values.

In contrast, ESR follows a slower kinetic profile. The observed moderate rise by day 3 supports its role in reflecting systemic inflammatory buildup, especially when interpreted alongside CRP. According to Kumar et al. (2022) [27], ESR alone may lack early sensitivity, but when tracked with CRP, it significantly strengthens the clinical assessment. Elevated ESR on day 3 in this dataset corresponds well with infections associated with high-virulence organisms, such as *Proteus vulgaris* and *Staphylococcus hominis* ssp. ESR values demonstrated greater variability compared with leukocyte counts, with a wider distribution across patients. This pattern likely reflects the slower kinetics and lower specificity of ESR, which captures sustained inflammatory processes rather than acute changes.

By day 7, ESR values peaked in many patients, highlighting its role as a delayed but persistent indicator of inflammation. Sereda et al. (2024) [28] emphasize that while ESR is less specific, its utility lies in monitoring long-term trends. The sustained elevation in our data may reflect either prolonged recovery or an undiagnosed low-grade infection.

Taken together, these graphical trends affirm the utility of combining CRP and ESR for postoperative monitoring. CRP provides rapid feedback on acute changes, while ESR offers insight into chronic or lingering inflammatory processes. Monitoring both markers dynamically, as done here, enables earlier and more accurate identification of potential complications, guiding timely interventions.

The limitations of the study include the relatively small number of cases, which limits the statistical applicability and generalizability of the conclusions. This was primarily due to the limited number of eligible arthroplasty procedures performed at our institution during the study period and the logistical complexity of performing standardized intraoperative microbiological sampling. Larger multicenter studies are required to validate these findings. The absence of randomization introduces a potential selection bias, and the lack of advanced molecular methods reduces the accuracy of biofilm detection. Nevertheless, the study provides a valuable factual basis and opens new directions for clinical and microbiological research [29].

Although often asymptomatic, intraoperative contamination can have significant clinical consequences through mechanisms such as biofilm formation, persistent inflammatory activation, and periprosthetic colonization. The data suggest that monitoring postoperative inflammatory markers, revising disinfection protocols, and individualizing antibiotic prophylaxis are key elements in preventing infectious complications in total arthroplasty. Several strategies may also contribute to reducing postoperative inflammatory responses and minimizing the risk of periprosthetic joint infection. Strict control of operating room traffic and limiting door openings can significantly reduce airborne microbial contamination. Optimized perioperative antibiotic prophylaxis tailored to the local microbiological spectrum may improve coverage against resistant organisms. Improved skin preparation protocols and adherence to sterile field management are critical for preventing endogenous contamination.

## 5. Conclusions

The presence of microorganisms in intraoperative samples reflects potential contamination of the surgical environment; however, this should not be directly equated with clinically significant infection. In this cohort, postoperative inflammatory markers—particularly CRP on day 3 and leukocyte count—were useful for monitoring the dynamics of the inflammatory response, although their specificity for infection remains limited. The results highlight the importance of integrating biological markers with clinical and microbiological data and underscore the need to distinguish between contamination, colonization, and true infection. Further large-scale studies are required to clarify the clinical significance of intraoperative contamination and to better define the role of inflammatory markers in the early detection of periprosthetic joint infection.

## Figures and Tables

**Table 1:** Shapiro–Wilk test for normality of inflammatory markers.

Variable	Shapiro–Wilk Statistic	*p*-Value	Distribution
CRP day 1	0.528229	2.26 × 10^−8^	Non-normal
CRP day 3	0.733958	2.92 × 10^−6^	Non-normal
CRP day 7	0.660069	1.71 × 10^−7^	Non-normal
ESR day 1	0.941568	0.075459	Normal
ESR day 3	0.949597	0.129479	Normal
ESR day 7	0.912284	0.011267	Non-normal

**Table 2:** Descriptive statistics of postoperative inflammatory markers.

Variable	Mean	Median	Std Dev	Q1 (25%)	Q3 (75%)
CRP day 1	72.01	70.38	7.19	66.21	78.36
CRP day 3	118.05	105.78	29.96	99.26	121.48
CRP day 7	113.06	101.48	35.74	94.52	112.27
ESR day 1	19.60	19.13	3.11	17.21	22.30
ESR day 3	26.48	26.28	5.37	22.50	29.04
ESR day 7	38.15	37.12	9.55	30.72	41.29

## Data Availability

The data that support the findings of this study are available from the corresponding author upon reasonable request.

## Abbreviations

PJI	Periprosthetic joint infection
CRP	C-reactive protein
ESR	Erythrocyte Sedimentation Rate
IDSA	Infectious Diseases Society of America
PCR	Polymerase Chain Reaction
IBM	International Business Machines
SPSS	Statistical Product and Service Solutions
